# Supplementary material for: Isolation and Quantification of Bacterial Membrane Vesicles for Quantitative Metabolic Studies Using Mammalian Cell Cultures
Source: Cells. 2023 Nov 21;12(23):2674. doi: 10.3390/cells12232674 (PMC10705164; doi:10.3390/cells12232674)
Supplement: Supplementary file 1 [file cells-12-02674-s001.zip › cells-2657349-supplementary.pdf]

ISOLATION AND QUANTIFICATION OF BACTERIAL MEMBRANE VESICLES FOR QUANTITATIVE METABOLIC STUDIES USING MAMMALIAN CELL CULTURES

Marcel Kretschmer[1,4,*], Julia Müller[1,4,*], Petra Henke[2], Viktoria Otto[3], Alejandro Arce Rodriguez[3], Mathias Müsken[5], Dieter Jahn[3,4], José Manuel Borrero-de Acuña[6], Meina Neumann-Schaal[2,4], and Andre Wegner[1,4]

[1]Department of Bioinformatics and Biochemistry, Technische Universität Braunschweig, Rebenring 56, 38106 Braunschweig, Germany
[2]Leibniz Institute DSMZ - German Collection of Microorganisms and Cell Cultures, Inhoffenstraße 7 B, 38124 Braunschweig, Germany
[3]Institute for Microbiology, Technische Universität Braunschweig, Braunschweig, Germany
[4]Braunschweig Integrated Center of Systems Biology (BRICS), Technische Universität Braunschweig, Rebenring 56, 38106 Braunschweig, Germany
[5]Central Facility for Microscopy, Helmholtz Centre for Infection Research (HZI), Inhoffenstraße 7, 38124 Braunschweig, Germany
[6]Department of Microbiology, Facultad de Biología, University of Sevilla, Av. de la Reina Mercedes 6, Sevilla, CP 41012, Spain
[*]These authors contributed equally

∗**Corresponding Author**

Andre Wegner
email: a.wegner@tu-bs.de

Keywords: bacterial membrane vesicles (BMVs) | membrane vesicles (MVs) | outer membrane vesicles (OMVs) | vesicle isolation | quantification | *Pseudomonas aeruginosa* | pathogen | SuhB | metabolism

SUPPLEMENTARY INFORMATION

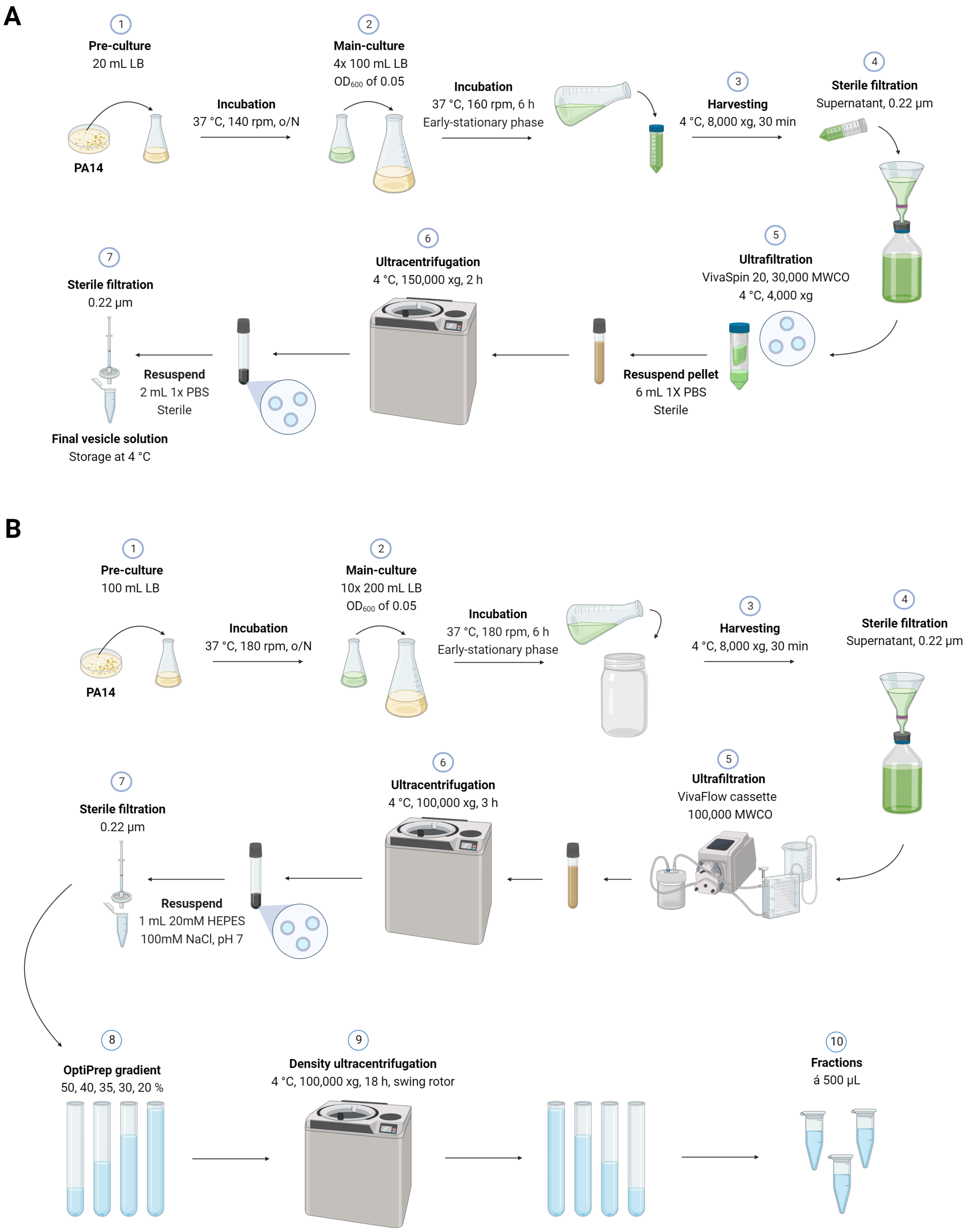

Figure S1: Isolation workflow of crude and pure *P. aeruginosa* BMVs. (**A**) Crude isolation of BMVs by ultrafiltration combined with ultracentrifugation: (1) Bacterial pre-cultures are grown overnight in LB broth (+ needed supplements like antibiotics). (2) Main cultures are inoculated with an OD_{600} of 0.05 and incubated until they reach the early-stationary phase of growth. (3) Bacterial cells are harvested and the culture supernatant filtered. (5) Ultrafiltration of sterile culture supernatants leads to concentrated material. (6) Membrane vesicles are pelleted by ultracentrifugation of concentrates and resuspended in a buffer. (7) Membrane vesicle solutions are sterile filtered and stored at 4 ℃. **(B)** Pure isolation of BMVs based on density gradient centrifugation. The first steps (1-7) are performed similar to the crude isolation method. Afterwards, an iodixanol density gradient is used to remove remaining contaminants and get the final vesicle fractions (8-10). Created with biorender.com.

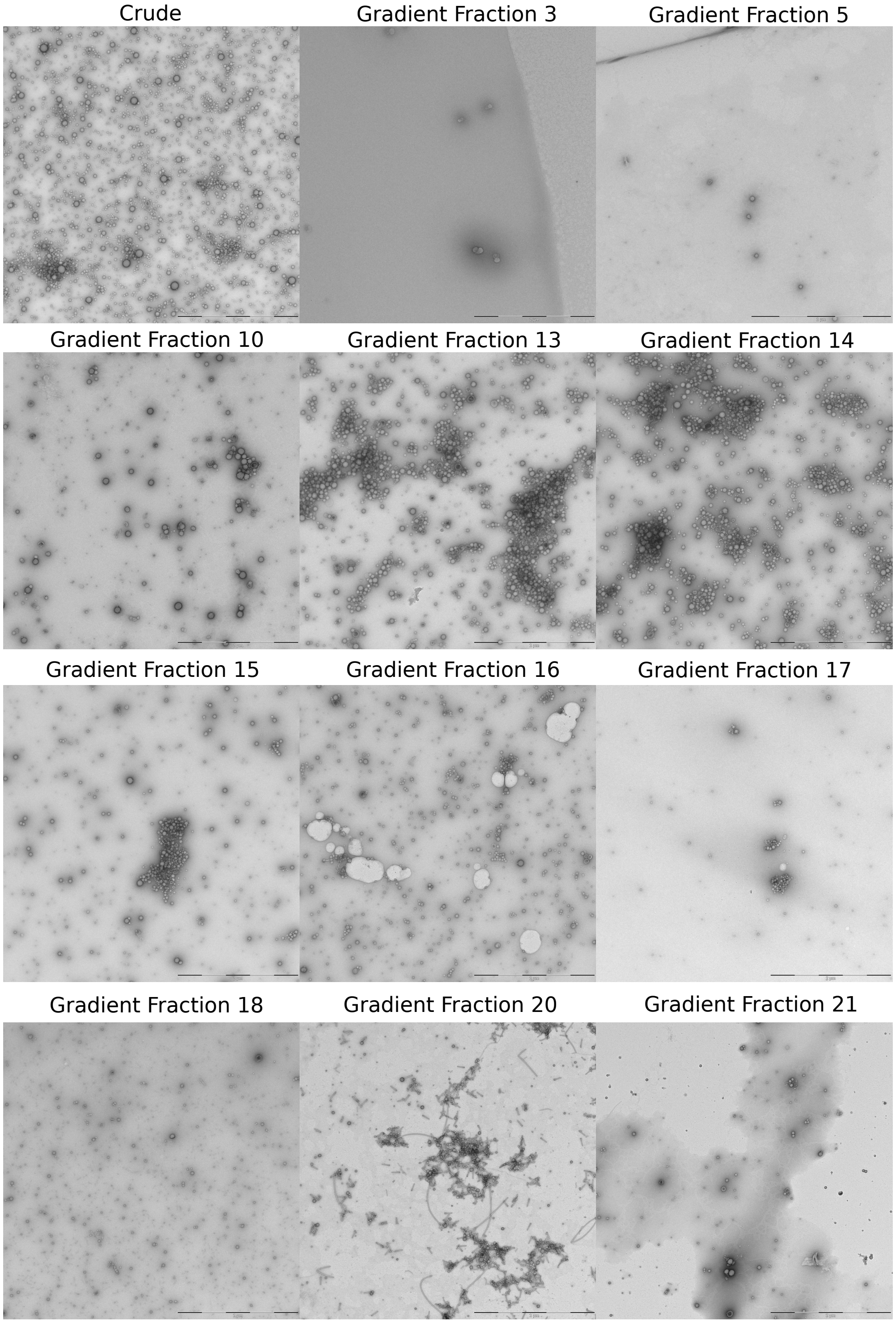

Figure S2: TEM images of selected negative stained *P. aeruginosa* BMVs. TEM images of negative stained crude and pure vesicles. Crude wild-type BMVs were isolated using the fast protocol. The wild-type vesicle fractions were isolated with the iodixanol-based density gradient centrifugation protocol. Scale bars = 2 µm.
